# Nutrient intake and nutritional status of newly diagnosed patients with cancer from the East Coast of Peninsular Malaysia

**DOI:** 10.1186/1756-0500-7-680

**Published:** 2014-09-30

**Authors:** Kavitha Menon, Shariza Abdul Razak, Karami A Ismail, Bhavaraju Venkata Murali Krishna

**Affiliations:** Advanced Medical and Dental Institute, Universiti Sains Malaysia, Bandar Putra Bertam, Kepala Batas 13200, Penang, Malaysia; School of Health Sciences, Universiti Sains Malaysia, Kota Bharu, Kelantan, Malaysia; School of Medical Sciences, Universiti Sains Malaysia, Kota Bharu, Kelantan, Malaysia

**Keywords:** Cancer, Nutritional status, Nutritional screening, Malnutrition, Anaemia

## Abstract

**Background:**

Cancer therapy in Malaysia primarily focuses on the clinical management of patients with cancer and malnutrition continues to be one of the major causes of death in these patients. There is a dearth of information on the nutrient intake and status of newly diagnosed patients with cancer prior to the initiation of treatment. The present study aims to assess the nutrient intake and status of newly diagnosed patients with cancer from the East Coast of Peninsular Malaysia.

**Methods:**

A cross-sectional study was conducted using a convenient sample of newly diagnosed adult patients with cancer (n = 70) attending the Oncology clinic, Hospital Universiti Sains Malaysia in the East Coast of Peninsular Malaysia. Information on socio-demographic characteristics, clinical status, anthropometry, dietary intake and biochemical data including blood samples was obtained.

**Results:**

The mean (SD) age, triceps skin fold (TSF), mid upper arm circumference (MUAC) and body mass index (BMI) of participants was 21.1(3.9) years, 17.6(7.9) mm, 24.1(5.5) cm, and 21.1(3.9) Kg/m^2^, respectively; 39% participants had BMI <18.5 Kg/m^2^. One-third of newly diagnosed patients with cancer were undernourished (i.e. women: MUAC <220 mm; men: <230 mm). The proportion (%) of participants with low haemoglobin (<120 g/L) and serum albumin (<38 g/dL) were 62% and 26%, respectively. The older women had significantly lower macro and micro nutrient intakes compared to men in the same age group (P <0.05).

**Conclusions:**

At the time of diagnosis, greater than one-third of patients with cancer from the East Coast of Peninsular Malaysia were underweight and undernourished. The majority of patients with cancer had poor micronutrient intakes; the older women had a poor macro and micronutrient intakes. Before the initiation of rigorous clinical management of patients with cancer, screening for nutritional status, subsequent nutrition counseling, and interventions are essential to improve their nutritional status; consequently, response to cancer therapy, survival and quality of life.

## Background

There has been an increase in the number of new cancer cases diagnosed worldwide, including Malaysia [1]. According to the current estimates, cancer is the fourth leading cause of death in Malaysia and about 27,000 newly diagnosed patients with cancer were registered in 2008 [2]. Malaysia has a National Cancer Control Programme (1997) in place to improve the quality of life of these patients through early diagnosis, appropriate cancer treatments, alternative therapies, and rehabilitation facilities [3]. However, the clinical care of patients with cancer focuses on the medical management with less attention on disease-related malnutrition that exists even at the time of diagnosis [4].

Patients with cancer have high nutrient requirements due to increased metabolic rate [5]. Tumor-induced pathophysiological changes alter the macronutrient metabolic pathways leading to increased protein catabolism, muscle protein degradation and elevated lipid oxidation [6]. Subsequently, cancer patients develop dysgeusia and poor appetite that adversely influence their food intake. In turn, reduced food intake coupled with altered metabolic changes lead to the onset of cancer cachexia with the classic clinical presentation of wasting, anorexia and systemic inflammation. As a result, disease-related malnutrition in patients with cancer often manifest as an early indicator of a rapidly progressing disease [7].

Although malnutrition is common in patients with cancer, is often overlooked in clinical settings [8]. The prevalence of disease-related malnutrition ranges from 20% to 80% in patients with cancer and, increases the risk of adverse outcomes, including poor prognosis [9, 10], response to treatment [11] and quality of life [12, 13]. Further, some studies have estimated that about 20% of head and neck and gastrointestinal patients with cancer die due to malnutrition than from cancer per se [9, 14]. The nutrient intakes and status are adversely affected during the course of cancer treatment; aggravates the pre-existing malnutrition to increase the risk of morbidity, longer hospitalization, cost of treatments and mortality [15].

On the contrary, nutritional screening prior to cancer treatments potentially identifies the existing malnutrition. With timely nutrition interventions prior to the initiation and during cancer therapy may be beneficial to reduce the cytotoxic effects and associated complications [16]. However, there is rarely any systematic nutritional screening of newly diagnosed patients with cancer in routine clinical practice in Malaysia; and the terminally ill patients with severe malnutrition or cachexia in critical stages of this disease are often referred to a dietitian. Nutritional counselling and interventions at this critical stage may have limited benefits compared to earlier referrals and timely interventions.

Information on the nutrient intakes and status of patients with cancer may benefit medical and paramedical staff to develop a multidisciplinary team approach to manage patients with cancer and to achieve optimum clinical outcomes. There is limited information on the prevalence of malnutrition in newly diagnosed patients with cancer from the East Coast of Peninsular Malaysia. Hence, the aim of this study was to assess the nutrient intake and status of newly diagnosed patients with cancer from the East Coast of Peninsular Malaysia attending the Oncology clinic of the Hospital Universiti Sains Malaysia; the primary cancer referral center for people from Kuala Terengganu, Pahang and Kelantan, the East Coast of Peninsular Malaysia.

## Methods

### Study design and participant recruitment

A cross-sectional study was conducted to assess the nutrient intake and nutritional status of newly diagnosed patients with cancer attending the Oncology unit, Hospital Universiti Sains Malaysia. The East Coast of Peninsular Malaysia has unique cultural practices; environmental and geographical distribution; and the population has a lower socio-economic status compared to other regions of Peninsular Malaysia. A convenient sample of patients with cancer (n = 70) aged between 20-70 years was recruited between December 2007 and December 2008 on their first visit to the Oncology clinic after diagnosis of cancer. Participants (i.e. patients with cancer) with comorbidities such as diabetes, hypertension, renal disorders, cardiovascular disorders, diseases that require modified dietary intakes were excluded. Participants with histopathologically confirmed cancer before undergoing any therapeutic interventions were recruited to the present study. The Human Research Ethics Committee at the Universiti Sains Malaysia approved the study. Informed consent was obtained from all participants prior to their recruitment to the study.

### Socio-demographic information, clinical data and anthropometry

Trained research assistants collected information on socio-demographic factors and clinical status using questionnaires. Data collected included age, gender, ethnicity (i.e. Malay, Chinese, Indian), annual household income, food habit (i.e. vegetarian or non-vegetarian) and clinical diagnosis (i.e. type of cancer, location of cancer and stage of cancer). Also, prior training was given to research assistants to collect anthropometric data using standard protocols; anthropometric measures such as height, weight, mid-upper arm circumference (MUAC), and triceps skin fold thickness (TSF) were obtained [17]. Participants were weighed in light clothing without shoes on a Seca digital weighing scale with a graduation of 0.1 Kg. The participant height was measured using a stadiometer; graduation 1.0 mm. The body mass index (BMI) was calculated from participant’s weight and height and participants of age <60 years were classified as per WHO classification [18]. The BMI of elderly participants (i.e. ≥60 years) was classified according to Lipschitz [19]. The MUAC was measured using a flexible steel metric tape to the nearest 1.0 mm while TSF was measured using a Harpenden skinfold caliper to the nearest 0.2 mm. A MUAC value <220 mm for women and <230 mm for males indicate grade 3 chronic energy deficiency [20]. The mean MUAC (cm) and TSF (cm) were used to calculate mid-arm muscle circumference (MAC) and corrected arm muscle area (AMA) using standard equations [21]. The corrected AMA value ≤21.4 cm^2^ for males and ≤21.6 cm^2^ for females were used to determine the risk of malnutrition [22].

### Biochemical parameters

Morning peripheral venous blood samples from participants were obtained in 2.0 ml EDTA vacutainers and 2.0 ml plain vacutainers (Becton Dickinson, Franklin Lakes, NJ) on their first visit to the Oncology clinic after cancer diagnosis. A complete blood cell count (CBC) was conducted at the Haematology laboratory and serum albumin was estimated at the Biochemistry laboratory attached to the Hospital Universiti Sains Malaysia, Kota Bharu.

### Nutrient intake assessment

Trained research assistants collected the dietary data using a modified interactive twenty-four hour recall [23] for a single day (i.e. the modified 24-hour interviewer based recall used visual aids, food models, food portions and probing questions to overcome the memory lapses of the respondents). Participants were asked for detailed descriptions and portions of foods and beverages consumed; recipes were collected whenever foods consumed were not available in the Malaysian food database. When the food weights were unavailable during data collection, foods were bought from the local restaurants and weighed using digital weighing scales to estimate the food weight. The food intakes (i.e. from the portion sizes recorded on the food recall form) were converted to food weights using the guidelines recommended by Gibson and Ferguson [23]. The food weights obtained using 24-hour recall were converted to nutrient intake estimates per day using Microsoft EXCEL programme based on the Malaysian food composition database [24]. Food items unavailable in the Malaysian food composition database were obtained from the Singapore food composition guide [25]. Information of any supplement use, food habit (i.e. vegetarian or non-vegetarian) and any special foods consumed were gathered through a separate questionnaire. Individual macro and micronutrient intakes were estimated and the mean intakes were compared between men and women. The nutrient intakes estimated include macronutrients (i.e. energy, carbohydrate, protein, fat); macrominerals (i.e. calcium and phosphorus); and micronutrients (i.e. iron, vitamin A, thiamine, riboflavin, niacin, and vitamin C). Information on nutrients such as folic acid, selenium, iodine, zinc, vitamin B_6_, vitamin B_12_ and the total fiber in the Malaysian food composition database is unavailable. The recommended nutrient intake for Malaysians (RNI) was used as the benchmark to assess the adequacy of energy intake and the distribution of energy from macronutrients such as carbohydrate, protein and fats [26]. Dietary adequacy of selected nutrient intakes was evaluated against the 77% recommended nutrient intakes for Malaysians as the estimated average requirements (EAR) for Malaysians was unavailable [27].

### Statistical analysis

Data processing and statistical analysis were carried out using Stata 12.0 (Stata Corporation, College Station, TX, USA). Participant characteristics were summarized using descriptive statistics such as mean, median, standard deviation, and proportions, for variables investigated. The chi-square test was used to investigate differences in the proportions of categorical variables; and the Fisher’s exact test was used when there were <5 observations. Differences in the mean values of biochemical parameters between genders (i.e. men vs women) were compared using independent t-test. Participants were divided into two groups based on their age group to 20-50 years (i.e. young adults) and 51-70 years (i.e. older adults) for dietary intake data analyses. The classification was performed to compare the mean nutrient intakes of participants in specific age groups (i.e. young adults and older adults) of Malaysian populations [25]. In addition, median and percentiles (i.e. 25^th^ and 75^th^) were reported for each nutrient. Mann-Whitney U-test was used to compare the nutrient intakes between men and women. The level of significance was set at P <0.05 for all analysis.

## Results

A total of 95 participants, who were on their first visit to the Oncology clinic after confirmed diagnosis of cancer, were approached; 70 participants completed the study (73.6% response rate). Of the 25 participants excluded, 14 failed to provide dietary data, and 11 had incomplete dietary recalls. Participants recruited to the study did not have any pre-existing co-morbidities.

The baseline characteristics showed that greater than half of the participants were <50 years of age, 60% were Malay, all were non-vegetarians and 80% consumed no supplements (Table 1). The median (min, max) income was RM 1000 (300, 4300) with a third earning less than RM 1500 per month. Participants had different types of cancer: cancers of the digestive organs (n = 22; 31.4%), breast (n = 13; 18.6%), thyroid (n = 9; 12.9%), respiratory system (n = 8; 11.4%), genitourinary (n = 6; 8.6%) and others including bone, cervix, head and neck, blood and bladder cancers (n = 12; 17.1%). The proportion of participants diagnosed with cancer at stage 1 and 2 were 33.3% and 42.0%, respectively; 18.9% participants were presented with late locally advanced cancer (i.e. Stage 3) and 5.8% in metastasized stage of disease (i.e. Stage 4).

**Table 1:** **Selected socio-demographic characteristics of newly diagnosed patients with cancer from the East Coast of Peninsular Malaysia**

Parameters	Overall (n = 70)	Men (n = 35)	Women (n = 35)	P-value
^a^Age	48 (20, 70)	49 (23, 70)	46 (20, 70)	
20-50 years	40 (57.1%)	20 (57.1%)	20 (57.1%)	^b^0.454
51-70 years	30 (42.9%)	15 (42.9%)	15 (42.9%)	
Gender	70 (100.0%)	35 (50.0%)	35 (50.0%)	-
Race				
Malay	60 (85.7%)	28 (80.0%)	32 (91.3%)	
Chinese	8 (11.5%)	7 (20.0%)	1 (2.9%)	^c^0.030
Others	2 (2.8%)	-	2 (5.8%)	
^a^Monthly household income (RM)^d^	1000 (300, 4300)	1000 (300, 4000)	700 (300, 4300)	^b^0.122
<RM 1500	43 (70.5%)	22 (66.7%)	21 (75.0%)	
RM 1500-3500	15 (24.6%)	9 (27.2%)	6 (21.4%)	^c^0.903
>RM 3500	3 (4.9%)	2 (6.1%)	1 (3.6%)	
Non-vegetarian	70 (100%)	35 (50%)	35 (50%)	-
Never used any supplements	56 (80%)	28 (80%)	28 (80%)	-

^a^Median (min, max); ^b^Two-sample t-test; ^c^Fisher's exact test; ^d^n = 61.

The mean anthropometric estimates suggest that men were taller than women (p < 0.05), greater than one-third of participants were underweight and about one-third were undernourished (Table 2). The mean CAMA were significantly higher for females compared to males (P < 0.001). Almost two-third of the participants were anaemic and one quarter was malnourished (i.e. serum albumin concentration <38 g/L) (Table 2). Twenty-seven percent of participants who were underweight (n = 19), had income below RM 1500; however, they were not different from the rest of the group (P = 0.094) (data not provided).

**Table 2:** **Anthropometric and biochemical measures of newly diagnosed patients with cancer from the East Coast of Peninsular Malaysia**

Parameters	Overall	Men	Women	P-value
Anthropometry^a,b^				
^c^Height (cm)	157.9 (8.9)	160.0 (8.3)	155.4 (9.1)	0.026
^c^Weight (Kg)	52.4 (10.8)	52.9 (9.7)	51.8 (11.9)	0.660
^c,d^BMI (Kg/m^2^)	21.1 (3.9)	20.5 (3.2)	21.7 (4.7)	0.244
^e^Under weight	26 (39%)	16 (46%)	10 (31%)	
^e^Normal	31 (46%)	16 (46%)	15 (47%)	^f^0.269
^e^Overweight/Obese	10 (15%)	3 (8%)	7 (22%)	
^c^Triceps skinfold thickness (mm)	17.6 (7.9)	16.2 (7.9)	19.1 (7.9)	0.135
^c^MUAC (cm)	24.1 (5.5)	23.4 (6.0)	24.7 (4.9)	0.324
^e,h^Undernourished	22 (32%)	9 (27)	13 (38)	^g^0.300
^c^CAMA (cm^2^)	51.1 (25.4)	30.9 (7.2)	71.4 (20.4)	<0.001
^e,i^Risk of malnutrition	5 (7)	4 (12)	1 (3)	^f^0.356
Biochemical parameters^a,b^				
^c^Haemoglobin (g/L)	120.4 (18.0)	123.9 (18.7)	117.1 (17.2)	0.232
^e,j^Anaemic	26 (62%)	14 (70%)	12 (55%)	^g^0.303
^c^Serum albumin (g/L)	38.7 (7.0)	37.7 (7.2)	39.8 (6.8)	0.362
^e^<38 g/L (Malnourished)	18 (26%)	8 (23%)	10 (29%)	^g^0.584

BMI- Body Mass Index; MUAC-Mid Upper Arm Circumference; CAMA: Corrected Arm Muscle Area.

^a^Number of participants: Height (Men: 35; Women = 32); Weight (Men = 35; Women = 34); MUAC, TSF (Men = 34; Women = 34); Haemoglobin (Men:22; Women:20); Albumin (Men: 20; Women:20); ^b^Mean (SD); ^c^T-test; ^d^For adults aged <60 years: Underweight: BMI ≤18.5 (Kg/m^2^); Normal: BMI >18.5 - ≤24.5 (Kg/m^2^); Overweight/Obese: BMI >25 (Kg/m^2^); For adults aged >60 years: Underweight: BMI ≤22 (Kg/m^2^); Normal: BMI >22 - ≤27 (Kg/m^2^); Overweight/Obese: BMI >27 (Kg/m^2^); ^e^Number (%); ^f^Fisher's exact test; ^g^Chi-square test; ^h^Undernourished: Women: MUAC <220 mm; Men <230 mm; ^i^Risk of malnutrition: Men: <21.4 cm2; Women: <21.6 cm2; ^j^Anaemia: Men: Hb < 130 g/L; Women: Hb <120 g/L.

Intakes of macronutrients and selected micronutrients are summarized in Table 3. The proportion of energy derived from carbohydrate, protein and fats for age group 20-50 years and 51-70 years were 59.0%, 16.1%, 24.5% and 53.8%, 17.1%, 26.7%, respectively. Except for the proportion of energy derived from carbohydrate in older adults, the macronutrient contributions to energy was within the recommendations of the Technical Subcommittee on Energy and Macronutrients recommendations for Malaysian Population and WHO recommendations for normal adults [28, 26]. The dietary intakes of carbohydrate (P < 0.05), protein (P < 0.001), phosphorus (P < 0.05), iron (P < 0.05), riboflavin (P < 0.001), and niacin (P < 0.001) were significantly higher in older men (i.e. 51-70 years) than women of the same age group. Except for a higher vitamin C intake in young adult women (P < 0.05), the dietary intakes of other nutrients of interest were similar in young men and women (i.e. 20-50 years). Overall, the median intakes of energy and many nutrients were below the recommended nutrient intakes for Malaysians for both age groups and genders.

**Table 3:** ^**a**^ **Median (25th, 75th percentiles) nutrient intakes of newly diagnosed cancer patients from the East Coast of Peninsular Malaysia (per day)**

Nutrients	RNI	Overall	20-50 yrs		RNI	Overall	51-70 yrs		
	Men, Women		Men (n = 20)	Women (n = 20)	Men, Women		Men (n = 15)	Women (n = 15)	Intake per 1000 KJ
Energy (Kcal)	10248-10332, 8400-9156	6271 (4813, 8064)	6791 (5145, 9463)	5615 (4523, 6808)	8442, 7476	5338 (4553, 7745)	7132 (5426, 8854)^b^	4721 (3818, 5246)	-
**Macronutrients**									
Carbohydrate (g)	-	193 (154, 283)	230 (163, 332)	187 (152, 249)	-	187 (134, 250)	235 (183, 273)^b^	137 (109, 196)	33.7 (29.3, 38.0)
% Energy from CHO	55-70%	59.0	58.2	59.8		53.8	54.9	52.9	
Protein (g)	62, 55	57 (36, 88)	69 (38, 96)	54 (35, 61)	59, 51	55 (36, 72)	72 (55, 101)^c^	37 (35, 56)	8.5 (7.4, 10.7)
% Energy from Protein	10-15%	16.1	16.1	16.1		17.1	19.7	14.5	
Fat (g)	-	42 (26, 58)	52 (25, 67)	37 (26, 47)	-	39 (27, 60)	49 (30, 62)	28 (25, 53)	6.7 (5.0, 8.8)
% Energy from Fat	20-30%	24.5	24.9	24.1		26.7	25.8	27.8	
**Macrominerals**									
Calcium (mg)	800, 800	264 (201, 391)	295 (194, 377)	250 (208, 452)	1000, 1000	249 (173, 404)	306 (156, 464)	217 (173, 301)	173.9 (133.5, 261.8)
Phosphorus (mg)	-	763 (528, 1402)	1175 (576, 1629)	720 (528, 854)	-	739 (579, 1147)	974 (716, 1231)^b^	579 (415, 895)	132.6 (103.5, 170.6)
**Micronutrients**									
Iron (mg)	14, 29	10.8 (6.7, 16.9)	11.2 (6.7, 16.6)	10.1 (7.0, 16.9)	14, 11	7.7 (5.6, 14.8)	13.9 (6.9, 18.1)^b^	6.5 (4.3, 10.6)	1.6 (1.1, 2.2)
RE (μg)	600	438 (288, 667)	467 (300, 667)	436 (288, 757)	600	573 (304, 857)	582 (350, 857)	467 (300, 667)	83 (50, 114)
Thiamine (mg)	1.2, 1.1	0.7 (0.5, 1.0)	0.7 (0.5, 1.4)	0.7 (0.5, 1.0)	1.2, 1.1	0.6 (0.5, 0.9)	0.8 (0.4, 1.3)	0.6 (0.5, 0.6)	0.1 (0.1, 0.2)
Riboflavin (mg)	1.3, 1.1	0.9 (0.6, 1.3)	1.0 (0.7, 1.4)	0.8 (0.4, 1.2)	1.3, 1.1	1.0 (0.7, 1.5)	1.5 (0.9, 1.7)^b^	0.7 (0.5, 1.2)	0.2 (0.1, 0.2)
Niacin (mg)	16, 14	9 (6, 14)	10 (6, 17)	8 (6, 13)	16, 14	6 (5, 12)	12 (6, 26)^c^	5.0 (4, 6)	1.2 (1.0, 2.2)
Vitamin C (mg)	70, 70	35 (20, 49)	25 (11, 43)^b^	43 (34, 57)	70, 70	38 (26, 60)	40 (26, 76)	38 (19, 59)	5.7 (3.9, 9.6)

RNI: Recommended Nutrient Intake; CHO: Carbohydrate; RE: Retinol equivalents; ^a^Median (25th, 75th percentiles); ^b^Significant difference between genders (Mann-Whitney U-test, P <0.05); ^c^Significant difference between age groups (Mann-Whitney U-test, P <0.001).

Inadequate intakes of protein and selected micronutrients of young and older adult participants are presented in Table 4. In all, about 95% of participants had inadequate calcium intakes below the EAR and almost 75% of older adult participants had poor vitamin C intake. Greater than half of participants had poor intakes of key micronutrients such as thiamine (70%), iron (66%), and niacin (64%); and almost half of them had poor riboflavin (49%) and retinol (47%) intakes. One-third participants had inadequate intakes of protein.

**Table 4:** ^**a**^ **Inadequate intakes of selected nutrients below the EAR in newly diagnosed patients with cancer from the East Coast of Peninsular Malaysia**

Nutrients	EAR ^b^	20-50 yrs	EAR ^b^	51-70 yrs	Overall
	Men, Women		Men, Women		
Protein (g)	42, 47	13 (33%)	45, 39	10 (33%)	23 (33%)
Calcium (mg)	660, 660	38 (95%)	770, 770	28 (93%)	66 (94%)
Vitamin C (mg)	54, 54	32 (80%)	54, 54	20 (67%)	52 (74%)
Thaimine (mg)	0.9, 0.8	27 (68%)	0.9, 0.8	22 (73%)	49 (70%)
Iron (mg)	10.8, 22	28 (70%)	10.8, 8.5	18 (60%)	46 (66%)
Niacin (mg)	12, 11	24 (60%)	12, 11	21 (70%)	45 (64%)
Riboflavin (mg)	1.0, 0.8	21 (53%)	1.0, 0.8	13 (43%)	34 (49%)
RE (μg)	462	21 (53%)	462	12 (40%)	33 (47%)

EAR: Estimated Average Requirement; ^a^Number (percentage); EAR calculated as 77% of the Recommended Nutrient Intakes for Malaysians.

## Discussion

The present study provides the first data on the nutrient intake and status of newly diagnosed patients with cancer from the East Coast of Peninsular Malaysia prior to the initiation of any therapeutic interventions. The current study suggests that about 40% of newly diagnosed patients with cancer were underweight, one-third were undernourished and two-thirds were anaemic. The older women (i.e. age 51-70 years) had a poor macro and micro nutrient intakes compared to young and older men. In addition, one-third of the study participants had inadequate intakes for protein, and the majority consumed diets poor in micronutrients.

The present study showed a high prevalence of pre-existing malnutrition in newly diagnosed patients with cancer, even prior to the initiation of cancer treatments. Malnutrition in the study participants may have been due to the systemic effects of the undiagnosed cancer, such as altered appetite and sensory functions, anorexia, cancer related discomforts that decrease the food intake leading to weight loss [29]. The prevalence of malnutrition in the current study participants were lower than that reported in cancer patients from other countries [30, 31]; the prevalence rates reported in these studies were estimated in cancer patients undergoing clinical interventions in the hospitals. Furthermore, the study participants were newly diagnosed with cancer and three-quarters of them were in the early stages (i.e. stage 1 & 2) of cancer. A high prevalence of malnutrition in patients with cancer undergoing therapy is unsurprising as cancer treatments have implications on the patient food intakes due to the associated side effects such as nausea, vomiting, loss of appetite, taste and smell alterations and anorexia [32]. Moreover, cancer treatments may develop or aggravate the anorexia, and without adequate nutritional support may trigger a series of catabolic changes that lead to weight loss and early cancer cachexia [33].

The median energy intakes of participants from the East Coast Malaysia were below the RNI for normal young and older Malaysians; the intake of energy was significantly lower in women than men of the same age group. There are several reasons that may have contributed to the lower energy intakes in patients with cancer: Firstly, the cancer-related pathophysiological changes lead to the onset of anorexia and taste changes that compromise the food intake [32]. Secondly, the physical activity levels are altered due to disease-related discomforts, pain and malignancy, which may reduce the appetite [34]. Thirdly, the psychological stress and trauma associated with disease diagnosis might lead to depression that influences the food intake of patients with cancer [35]. The older women may have lower food intakes than men of the same age, which may precipitate in lower energy intakes, especially when they have less access to nutritious foods, living alone or have poor socio-economic background [36]. The energy requirements of patients with cancer are determined by the stage of cancer, the primary location of the cancer, physical activity levels including whether ambulatory or bed ridden [34].

The macronutrient intakes of newly diagnosed young adult patients with cancer (i.e. 20-50 years) were just within the range set for normal healthy individuals [26, 28]. Conversely, the energy derived from carbohydrates (i.e. 55-75% of energy) and the protein intake (i.e. <60% of RNI) in older adults was below the adequate levels of intakes [28], probably due to the implications of undiagnosed cancer. The nutrient requirements (i.e. marco and micro nutrients) of patients with cancer are higher than for normal healthy individuals to meet the increased metabolic turnover and to prevent disease-related wasting and malnutrition leading to cancer cachexia [6]. In addition, young adults with cancer should consume micronutrient dense diets to improve their intakes of vitamin C, iron, riboflavin and vitamin A. In older adults, the lower nutrient intakes could be attributed the dual burden of cancer-induced and age-related physiological changes that decrease the appetite and food intakes [36, 37]. The factors that contribute to decreased nutrient intake in these older participants may include low physical activity levels, poor appetite, apathy, altered taste and sensory functions, poor memory, co-morbidities, local tumor effects, discomforts, and altered metabolic changes [38]. The poor macro and micronutrient intakes of older women strongly indicate the need for special nutritional interventions, support and care prior to the initiation of cancer treatments. A third of undernourished participants in the current study at the time of diagnosis require higher intakes of energy and protein; and specific personalized nutritional interventions to improve their body weight and lean muscle mass.

The prevalence of inadequate micronutrient intakes was higher (>50%) in the study participants for many of the micronutrients investigated; consequently, a greater proportion had anaemia. In the current study, we have used an EAR cut-off point (i.e. <77% of RNI) to assess the risk of inadequate intakes of nutrients, which is lower than the recommended nutrient intake (RNI), however, the proportion of participants with inadequate intakes of micronutrients remained high. The use of RNI as cut-off point to estimate the prevalence of inadequate intakes of nutrients may overestimate the proportion of participants with inadequate intakes [23, 27]. Furthermore, with such poor micronutrient intakes it is likely that many of the study participants may have co-existing micronutrient deficiencies that may adversely influence cancer treatments and associated clinical outcomes [6].

Cancer-related pathophysiological processes together with reduced intakes of micronutrient dense foods might potentially induce a poor micronutrient status in patients with cancer. Evidence suggests that the patients with cancer in general have a lower micronutrient status of vitamin A, B, C, D, E, selenium and zinc, otherwise known as antioxidants compared to healthy individuals [6, 39]. Unless addressed prior to the initiation of cancer treatments, the micronutrient deficits may exacerbate the risk of complications after surgery, depression, and compromised immune competence that influence the clinical outcomes and quality of life of cancer patients [4]. The poor micronutrient intakes among the majority of the study participants mandate improvements in their diet quality with micronutrient dense foods; in cases where the food intake is compromised micronutrient supplementation through enteral or parenteral route is warranted.

Although recommendations for systematic evaluation of nutritional status of cancer patients using standardized protocols exist, in routine clinical practice it is widely neglected [8, 34]. The current study findings were surprising, as often a high prevalence of malnutrition was reported in hospitalized inpatients [10] or inpatients undergoing cancer treatments. Evidence suggests that nutrition interventions and nutrition counseling of patients with cancer improved response and tolerance to treatments, nutritional status, and quality of life [16]. We suggest a nutritional screening of cancer patients on their first visit to the Oncology clinic for treatments after diagnosis. In addition, we recommend regular nutritional interventions in malnourished cancer patients with systematic nutritional surveillance during treatments be made mandatory in Malaysia to reduce the risk of preventable morbidity and mortality due to cancer cachexia. Furthermore, vulnerable groups including older women should be given additional nutritional support, as they have the dual burden of the disease and ageing that may exacerbate the risk of malnutrition.

The limitations of the current study include the use of a single interactive 24-hour dietary recall, as an additional food recall would increase the respondent burden, thus, less precise estimates of nutrient intakes. Secondly, the 24-hour recall method of dietary intake data collection is subjective and dependent on the memory of the participants; however, we used a modified interactive dietary recall method to elicit more reliable dietary information. The use of single modified interactive 24 hour dietary recall is adequate to estimate the mean nutrient intakes of groups [23]. Thirdly, day-to-day variations in the nutrient intakes were unadjusted as the dietary intake data was collected in a single day. Nevertheless, the poor nutrient intakes reported in the current study participants were also reflected in their anthropometric and biochemical parameters. Fourthly, the Malaysian food composition database used for the dietary data analyses is out-of-date and thus, the changes in nutrient composition of foods over the time (i.e. post 1997) may not have adequately captured. However, the recent food composition databases developed with nutrient information on Malaysian foods continued to rely on the same Malaysian food composition database [40]; the use of this database for nutrient analysis was inevitable.

## Conclusion

Malnutrition in cancer patients is a concern in Malaysia and worldwide. According to our findings, one-third of patients with cancer from the East Coast of Peninsular Malaysia were malnourished at the time of diagnosis, greater than one-third were undernourished and two-thirds were anaemic. The proportion of energy derived from the macronutrients of young adult patients’ diets were within the normal ranges, however, was lower than their energy requirements. The older women with cancer had poor energy, macro and micronutrient intakes. The micronutrient intakes of both young and older patients with cancer were inadequate. A paradigm shift in the routine clinical practice is essential to incorporate early nutritional screening to identify the malnourished patients with cancer prior to the initiation of cancer treatments. The identified malnourished patients should be given nutritional counseling and interventions to correct the pre-existing nutritional inadequacies to improve their nutritional status, response to treatment, clinical outcomes, and quality of life.

## Abbreviations

AMA: Arm muscle area
BMI: Body mass index
CI: Confidence interval
MUAC: Mid upper arm circumference
MUC: Mid-upper arm circumference
SA: Serum albumin
SD: Standard deviation
TSF: Triceps skinfold thickness.
